# Boron delivery agents for neutron capture therapy of cancer

**DOI:** 10.1186/s40880-018-0299-7

**Published:** 2018-06-19

**Authors:** Rolf F. Barth, Peng Mi, Weilian Yang

**Affiliations:** 1 Department of Pathology, The Ohio State University, 4132 Graves Hall, 333 W. 10th Ave, Columbus, OH 43210 USA; 2 Department of Radiology, Center for Medical Imaging, State Key Laboratory of Biotherapy and Cancer Center, West China Hospital, Sichuan University and Collaborative Innovation Center for Biotherapy, Chengdu, Sichuan 610041 P. R. China; 3 Present Address: Neurosurgery and Brain and Nerve Research Laboratory, The First Affiliated Hospital of Suzhou University, Suzhou, Jiangsu 215004 P. R. China

**Keywords:** Boron delivery agents, Neutron capture therapy, Brain tumors, Head and neck cancer, Melanoma

## Abstract

Boron neutron capture therapy (BNCT) is a binary radiotherapeutic modality based on the nuclear capture and fission reactions that occur when the stable isotope, boron-10, is irradiated with neutrons to produce high energy alpha particles. This review will focus on tumor-targeting boron delivery agents that are an essential component of this binary system. Two low molecular weight boron-containing drugs currently are being used clinically, boronophenylalanine (BPA) and sodium borocaptate (BSH). Although they are far from being ideal, their therapeutic efficacy has been demonstrated in patients with high grade gliomas, recurrent tumors of the head and neck region, and a much smaller number with cutaneous and extra-cutaneous melanomas. Because of their limitations, great effort has been expended over the past 40 years to develop new boron delivery agents that have more favorable biodistribution and uptake for clinical use. These include boron-containing porphyrins, amino acids, polyamines, nucleosides, peptides, monoclonal antibodies, liposomes, nanoparticles of various types, boron cluster compounds and co-polymers. Currently, however, none of these have reached the stage where there is enough convincing data to warrant clinical biodistribution studies. Therefore, at present the best way to further improve the clinical efficacy of BNCT would be to optimize the dosing paradigms and delivery of BPA and BSH, either alone or in combination, with the hope that future research will identify new and better boron delivery agents for clinical use.

## Background

**Fig. 1:**
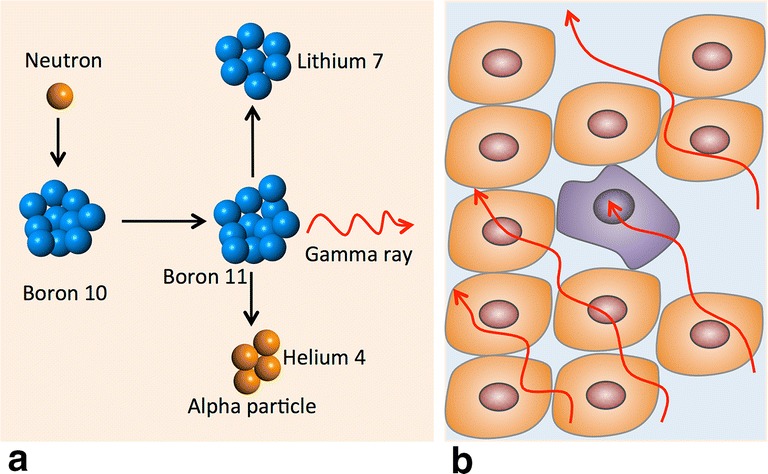
Boron neutron capture therapy is based on the nuclear capture and fission reactions that occur when non-radioactive boron-10, a constituent of natural elemental boron, 80% of which is in the isotopic form of ^11^B and 20% as ^10^B, is irradiated with low-energy (0.025 eV) thermal neutrons or, alternatively, higher-energy (10,000 eV) epithermal neutrons. The latter become thermalized as they penetrate tissues. The resulting ^10^B(n,α)^7^Li capture reaction yiels high linear energy transfer (LET) α paricles (stripped down helium nuclei [^4^He]) and recoiling lithium-7 (^7^Li) atoms (**a**).  A sufficient amount of ^10^B must be delivered selectively to the tumor (~ 20–50 μg/g or ~ 10^9^ atoms/cell) in order for BNCT to be successful (**b**). A collimated beam of either thermal or epithermal neutrons must be absorbed by the tumor cells to sustain a lethal ^10^B(n,α)^7^Li capture reaction. Since the α paricles have very short pathlengths in tissues (5–9 μm), their destructive effects are limite to boron-containing cells. In theory, BNCT provides a way to selectively destroy malignant cells and spare surrounding normal tissue if the required amounts of ^10^B and neutrons are delivered to the tumor cells.

Boron neutron capture therapy (BNCT) is based on the nuclear capture and fission reactions that occur when the stable isotope boron-10 (^10^B) is irradiated with either low-energy (0.025 eV) thermal neutrons or, for clinical studies, epithermal neutrons (10,000 eV), which become thermalized as they penetrate tissue. This results in the production of high-linear energy transfer (LET) alpha (α) particles (^4^He) and recoiling lithium-7 (^7^Li) nuclei (Fig. 1a). In order to be successful, ~ 20 μg/g of ^10^B per weight of tumor must be selectively delivered to the tumor cells (~ 10^9^ atoms/cell), and enough neutrons must be absorbed by them to sustain a lethal ^10^B(n, α)^7^Li capture reaction [1]. Since α particles have very short pathlengths (5–9 μm) their destructive effects are limited to boron-containing cells (Fig. 1b). In theory, α particles can selectively destroy tumor cells and spare adjacent normal cells. Clinical interest in BNCT has focused primarily on high grade gliomas [2–5], patients with recurrent tumors of the head and neck region [6–13] who have failed conventional therapy, and a much smaller number of patients with cutaneous [14–17] or extra-cutaneous [18] melanomas. Because BNCT primarily is a biologically, rather than a physically, targeted type of particle radiation therapy, it should be possible to selectively destroy tumor cells infiltrating normal tissue. The requirement, however, is that sufficient amounts of ^10^B and thermal neutrons are delivered to the site of the tumor. Up until 2014, the source of these neutrons has been specially designed nuclear reactors, but recently a number of companies in Japan [19] and the United States [20] have fabricated accelerator-based neutron sources, several of which are either being or will be evaluated in Phase I/II clinical trials.

In this review, we will focus on the two drugs that have been used clinically for BNCT and their limitations, as well as a variety of low and high molecular weight boron delivery agents that have been evaluated in vitro and in experimental animal tumor models, but have not been evaluated clinically. Interested readers are referred to several more comprehensive reviews for more detailed information relating to boron delivery agents [21–24].

## General requirements for boron delivery agents

The most important requirements for a BNCT delivery agent are: (1) low intrinsic toxicity; (2) high tumor uptake (~ 20–50 μg ^10^B) and low normal tissue uptake, ideally with a tumor:normal tissue and tumor:blood boron concentration ratios of > 3:1; and (3) relatively rapid clearance from blood and normal tissues, and persistence in tumor for at least several hours during neutron irradiations. Approximately 50 years ago, research on the development of boron-containing delivery agents for BNCT began in the laboratory of Albert Soloway and his co-workers at the Massachusetts General Hospital in Boston. A large number of low molecular weight boron compounds were synthesized, from which the first second-generation compound emerged, a polyhedral borane anion, first synthesized by Miller et al. [25], sodium mercaptoundecahydro-*closo*-dodecaborate (Na_2_B_12_H_11_SH), commonly known as sodium borocaptate or BSH [26]. BSH first was used clinically by Hatanaka [2, 27] and Nakagawa [3] in Japan, and by Sauerwein and his research team in Europe [28, 29] in a Phase I/II clinical trial in Petten, The Netherlands, to treat patients with high grade gliomas.

A second boron compound, first synthesized by Snyder et al. in 1958 [30], was introduced by Mishima and co-workers in Japan, a boron-containing amino acid (L)-4-dihydroxy-borylphenylalanine, known as boronophenylalanine or BPA [14, 15, 31]. Based on the assumption that BPA would preferentially be taken up by melanin-synthesizing cells, it initially was used to treat several patients with cutaneous melanomas by injecting it perilesionally [14, 15, 31]. Experimental data of Coderre et al. [32] at the Brookhaven National Laboratory in the United States demonstrated that BPA also was taken up by other histologic types of tumors, including a rat brain tumor, the 9L gliosarcoma. Based on this observation, BPA, as a fructose complex (BPA–F) which significantly increased its water solubility [33], very quickly entered into clinical use for the treatment of patients with high grade gliomas. A number of clinical trials were initiated, first in the United States [34, 35] and subsequently in Finland [36, 37], Sweden [38, 39] and Japan [4, 5, 40–42], and these demonstrated that BPA was therapeutically more effective than BSH. It subsequently became the drug of choice for clinical BNCT of patients with high grade gliomas [2–5] and recurrent tumors of the head and neck region [6–13, 43]. Interested readers are referred to two recent reviews that discuss the clinical results obtained using BNCT to treat brain and head and neck tumors [44, 45].

The major problem with both BSH and BPA is the significant variability in tumor uptake, especially in brain tumors. This was clearly demonstrated by Goodman et al. [46]. in a biodistribution and pharmacokinetic study involving 20 patients with high grade gliomas. Tumor boron concentrations varied both within different regions of the tumor, as well as among patients who received the same dose of BSH. Similar variability was reported by Koivunoro et al. [47] in a group of 98 patients with gliomas who received BPA-F, although the blood and estimated normal brain boron concentrations were in a much narrower range. This variability in the tumor uptake of BPA and BSH most likely was due to the marked and complex intratumoral histologic, genomic, and epigenomic heterogeneity within high grade gliomas [48], as well as intertumoral variability from one patient to another. Experimental animal studies carried out by Barth and Yang and their co-workers using the F98 rat glioma model revealed similar variability in tumor boron concentration for both BSH and BPA in glioma-bearing rats. This suggested that the broad range in mean survival time (MST) following BNCT was a consequence of the variability in tumor uptake and microdistribution [49–52]. Similar variability also has been described in a nude rat model for neutron capture therapy of intracerebral melanoma [53].

## Third-generation boron delivery agents

Since neither BSH nor BPA adequately fulfills the criteria indicated in the preceding section on general requirements, there has been a pressing need to develop new boron delivery agents. With the development of improved synthetic techniques and an increased awareness of the requisite biochemical properties, a number of new boron delivery agents have emerged. The major challenge for their development has been the requirement for selective tumor cell targeting and the delivery of therapeutic concentrations of boron with minimal normal tissue uptake and retention. The effective killing of glioblastoma cells in the presence of normal brain tissue represents an even greater challenge than for malignancies at other anatomic sites. This is due to an additional biological impediment, the blood–brain barrier (BBB) [54, 55], which effectively excludes agents with molecular weights greater than 200 Da, and the highly infiltrative properties of glioma cells and their genomic heterogeneity.

**Fig. 2:**
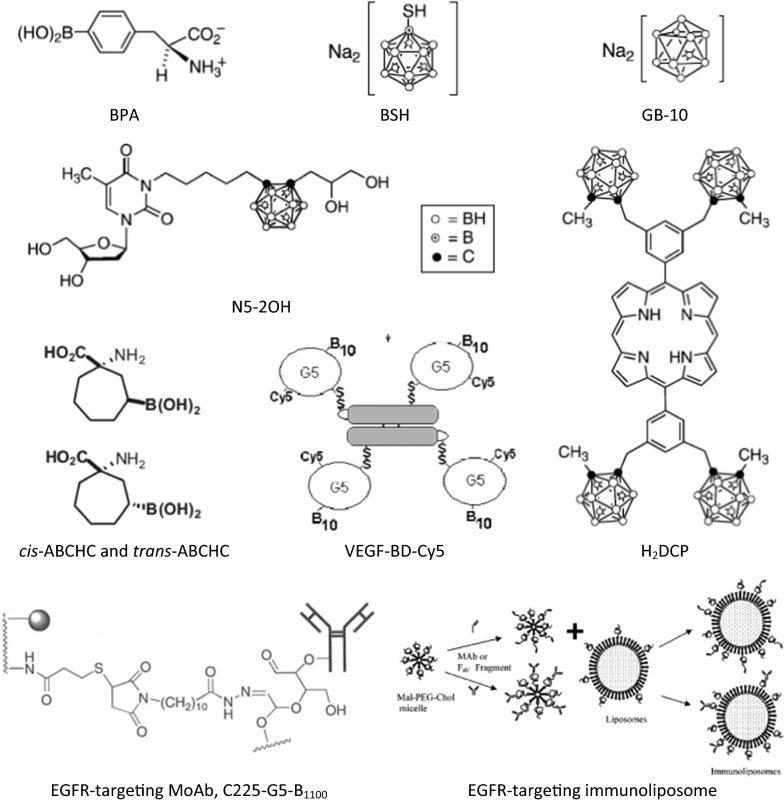
Some low- and high-molecular weight boron delivery agents (with the exception of #3) that have been investigated by Barth et al. (1) BPA (boronophenylalanine, Na_2_^10^B_10_H_10_) and (2) BSH (sodium borocaptate, Na_2_^10^B_12_H_11_SH, undecahydro-mercapto-*closo*-dodecaborate) are the only two drugs in clinical use. (3) GB–10 (sodium decaborate, Na_2_B_12_H) has been used in only a few animal studies; although at one time it had an approved U.S. Food and Drug Administration (FDA) Investigational New Drug designation (IND), it never has been used clinically. (4) N5-2OH (3-[5-{2-(2,3-dihydroxyprop-1-yl)-*o*-carboran-1-yl}pentan-1-yl] thymidine) is a carboranyl thymidine analogue (CAT) that yielded promising results in the RG2, but not the F98, rat glioma models following intracerebral convection-enhanced delivery (i.c. CED). (5) *cis*-ABCHC and *trans*-ABCHC (1-amino-3-borono-cycloheptanecarboxylic acid) as a racemic mixture is an unnatural amino acid that has in vivo uptake comparable to BPA in the B16 melanoma model, but far superior tumor:blood boron concentration ratios compared with BPA. (6) VEGF-BD-Cy5 is a heavily boronated vascular endothelial growth factor (VEGF) linked to Cy5 for near infrared imaging of the construct. (7) H_2_-DCP (di [3,5-(*nido*-carboranylphenyl) tetra-benzoporphyrin]) is one of a group of carboranyl porphyrins containing multiple carborane clusters, which show high in vitro cellular uptake. In vivo BNCT following i.c. CED yielded survival data comparable to that of intravenously administered BPA (8) C225-G5-B_1000_ is a heavily boronated form of the monoclonal antibody cetuximab that specifically targets the human epidermal growth factor receptor (EGFR), which has been used for BNCT of the F98_EGFR_ rat glioma. (9) EGFR-targeting, boron-containing immunoliposomes with cetuximab as the targeting moiety

**Fig. 3:**
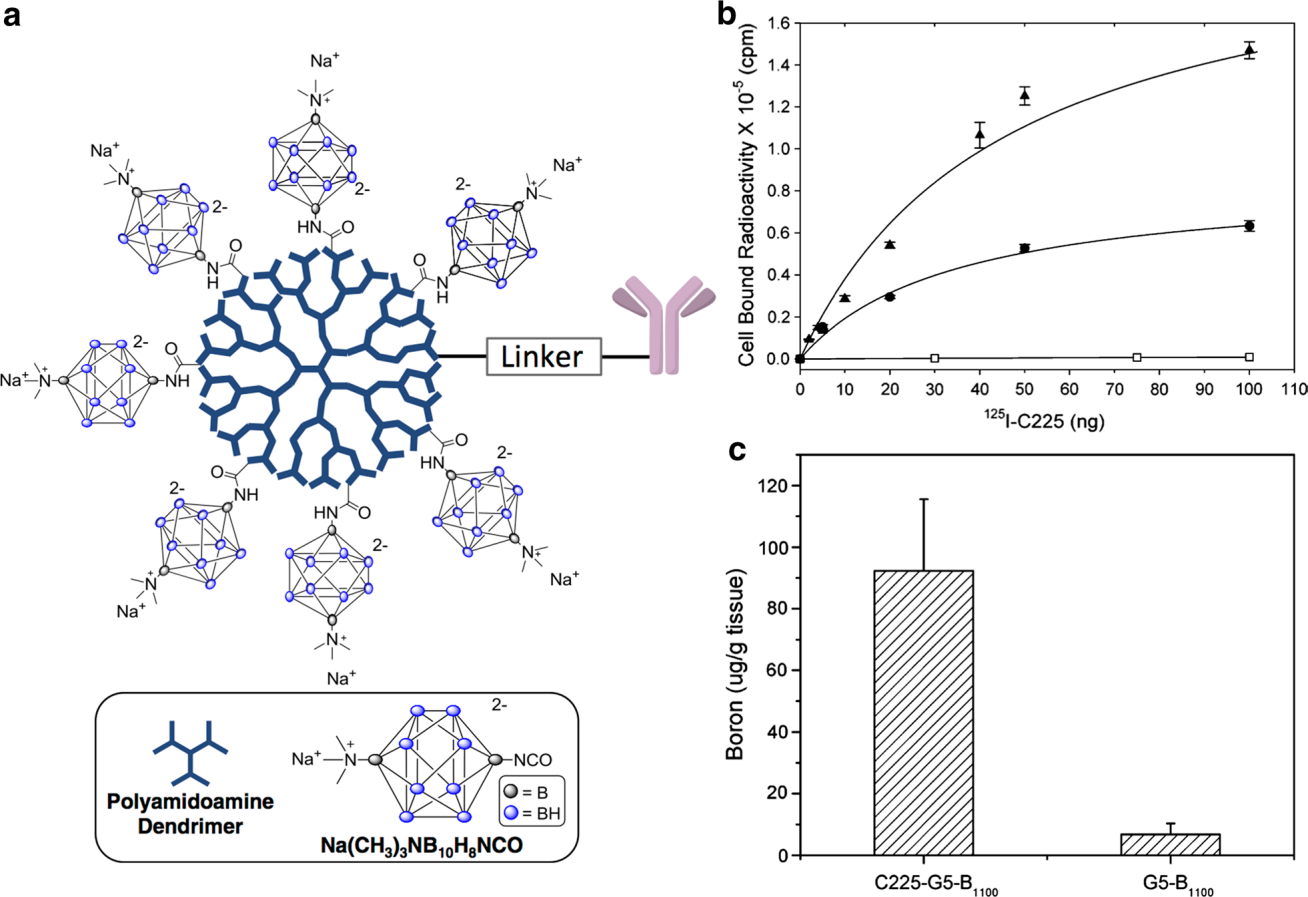
BSH-dendrimer conjugates for BNCT. **a** Conjugation scheme for the linkage of a boron-containing dendrimer to cetuximab; **b** Cellular binding of cetuximab. Varying amounts (5 − 100 ng) of ^125^I-cetuximab were incubated at 4 °C for 90 min with cells expressing wild-type EGF receptors (F98_EGFR_) (black up-pointing triangle), mutant EGFRvIII receptors (F98_EGFRvIII_) (black circle), and receptor-negative parental cells (F98_WT_) (white square). **c** Boron neutron capture therapy effect of BSH-polymer conjugation on colon 26 subcutaneous tumor-bearing BALB/c mice. Reproduced with permission. Copyright 2004, ACS [150]

Recent efforts to improve the selectivity of boron delivery agents has involved incorporating them into tumor-targeting moieties, such as unnatural amino acids, polyamines, peptides, proteins, antibodies, nucleosides, sugars, porphyrins, liposomes and nanoparticles [44]. A partial list of third generation boron delivery agents of low and high molecular weight is summarized in Table 1 and shown in Fig. 2. Among the low molecular weight boron delivery agents are boronated natural amino acids (i.e. BPA derivatives with higher percentage of boron by weight), as well as boronated derivatives of other amino acids such as aspartic acid, tyrosine, cysteine, methionine and serine [56–58]. Boron-containing unnatural amino acids also have been investigated because of their higher metabolic stability compared with the natural ones. The boronated derivatives of 1-aminocyclobutane-1-carboxylic acid (ABCHC) and 1-amino-3-boronocyclo-pentanecarboxylic acid (ABCPC) are examples of such compounds [57–60] (Fig. 2). Higher tumor and tumor:brain boron concentration ratios were obtained with ABCPC, but the tumor:blood ratios were comparable to that of BPA [61]. Unfortunately, no further animal studies have been carried out at the time of this writing on this promising class of compounds. Boron-containing linear and cyclic peptides conjugated to sodium borocaptate have been investigated because they are usually non-immunogenic, easy to synthesize, and often show low toxicity and high tissue penetrating properties [62]. Of particular interest are peptide ligands for over-expressed receptors on tumor cells, such as the vascular endothelial growth factor receptor (VEGFR) [63] (Fig. 2), somatostatin receptors and the epidermal growth factor receptor (EGFR and EGFR_VIII_) [64–66] (Figs. 2, 3). However, the major problem relating to VEGF as a targeting moiety is that it would require repeated applications of BNCT to be effective. EGFR on the other hand is variably expressed on glioma cells either in its wildtype form or its mutant variant, EGFR_VIII_.

**Table 1:** Examples of new low- and high-molecular weight boron delivery agents currently under evaluation

Boric acid [139]	Boronated VEGF [64]
Boron-containing immunoliposomes [101, 103] and liposomes [90, 91, 93, 94, 102, 104, 105]	Boronated unnatural amino acids [57, 61–64]
	Boron-containing nanoparticles [140–142]
Boron-containing Lipiodol [143–145]	Carboranyl nucleosides [70, 146]
Boron nitride nanotubes [147–149]	Carboranyl porphyrazines [139]
Boronated co-polymers [85, 86]	Carboranyl thymidine analogues [70–72]
Boronated cyclic peptides [62]	Decaborone (GB10) [131, 143]
Boronated DNA intercalators [77]	Dodecaborate cluster lipids and cholesterol derivatives [144]
Boronated EGF [82, 83] and anti-EGFR MoAbs [67–69, 150]	Dodecahydro-closo-dodecaborate clusters [144]
Boronated polyamines [147, 151]	Linear and cyclic peptides [65]
Boronated porphyrins [74–78]	Polyanionic polymers [86]
Boronated sugars [152]	Transferrin-polyethylene glycol liposomes [140]

The delivery agents are listed alphabetically and not in any order indicating their potential usefulness for BNCT. None of these agents have been evaluated clinically

*BNCT* boron neutron capture therapy, *EGF* epidermal growth factor, *EGFR* epidermal growth factor receptor, *MoAbs* monoclonal antibodies, *VEGF* vascular endothelial growth factor

Boron-containing purines, pyrimidines, thymidines, nucleosides and nucleotides also have been investigated as BNCT delivery agents, in particular 3-carboranyl thymidine analogues (3CTAs), which specifically target thymidine kinase-1 (TK1)-expressing tumor cells [67–69]. For example, in vitro studies of the thymidine derivative designated N5–2OH (Fig. 2) demonstrated selective tumor uptake, a high rate of phosphorylation and low toxicity [67], which led to in vivo biodistribution and BNCT studies in brain tumor–bearing rats. Convection enhanced delivery (CED), by which therapeutic agents are delivered directly to the brain and completely bypass the BBB [70], has been an effective way to deliver some boron compounds [68, 71] and high molecular weight bioconjugates to brain tumor–bearing rats [64–66]. CED of N5–2OH to rats bearing intracerebral RG2 gliomas was effective for the selective delivery of therapeutic concentrations of boron to tumors with very high tumor:brain and tumor:blood ratios and without any concomitant toxicity [68]. Following BNCT, a significant prolongation in the MST of tumor-bearing rats was observed [68]. However, similar studies carried out using the almost identical F98 rat glioma, which also expressed amplified TK1, only produced a modest increase in MST [72], suggesting that N5–2OH may not be as effective as a boron delivery agent as was originally thought [68].

Boron-containing porphyrin derivatives (porphyrins, chlorins, bacteriochlorins, tetrabenzoporphyrins, and phthalocyanines) have been studied extensively due to their low toxicity and natural affinity for tumors [73–75]. Examples of such compounds are BOPP [75], CuTCPH [21], and H_2_DCP [71] (Fig. 2). Porphyrin derivatives have been shown to deliver therapeutic amounts of boron to tumor bearing mice and rats, but as reported by Kawabata et al., this may not be localized in tumor cells [71]. In vivo biodistribution studies, carried out 24 h following intracerebral administration by means of CED to F98 glioma bearing rats, revealed unusually high tumor boron concentrations (~ 100 µg/g). Surprisingly, the MST 5-6 weeks following tumor cell implantation were very similar to those obtained using BPA, which attained much lower boron concentrations. Histologic examination of the brains of rats that received the boronoporphyrin compounds, followed by BNCT, revealed that they were localized in macrophages rather than tumor cells, thereby providing an explanation for the much lower than expected MST [71]. Further synthetic studies will be required to design porphyrin compounds that would have decreased affinity for macrophages and increased tumor cell uptake.

Other boron-containing DNA binding molecules, including alkylating agents, DNA intercalators, minor-groove binders and polyamines, have been investigated [76]. For example, derivatives of aziridines, acridines, phenanthridines, various Pt(II) complexes and carboranyl polyamines have been described [22–24]. These compounds sometimes show low tumor selectivity and significant toxicity, in part due to their multiple cationic charges and/or ability for binding to DNA of normal cells. Boron-containing sugars, including derivatives of glucose, mannose, ribose, galactose, maltose and lactose, also have been investigated [77]. This class of molecules usually have low toxicity, but also unfortunately low tumor uptake, in part due to their hydrophilicity and rapid clearance from tissues.

Among the high molecular weight boron delivery agents, monoclonal antibodies (MoAbs), polymers, dendrimers, liposomes and nanoparticles have been the most intensively studied. MoAbs are a very promising class of tumor-targeting agents due to their high specificity for molecular targets such as EGFR and EGFR_vIII_ [65, 66] and the ligands EGF [78] and VEGF [63]. Extensive studies have been carried out by Barth, Wu and Yang and their co-workers using a heavily boronated precision dendrimer with five dendritic generations that has been linked by means of heterobifunctional reagents to the EGFR targeting MoAb cetuximab (Erbitux™) [65], the EGFR_vIII_ targeting MoAb L8A4 [64] or EGF [79] itself (Fig. 3). These bioconjugates were administered intracerebrally by means of CED to rats bearing receptor positive F98 gliomas that have been transfected with the human gene encoding EGFR or EGFR_vIII_ (F98_EGFR_ or F98_EGFRvIII_) [64–66, 79, 80]. The best survival data were obtained in F98_EGFR_ glioma bearing rats when these bioconjugates were combined with intravenous administration of BPA, yielding a two to threefold increase in MST compared to irradiated controls [64–66, 80]. However, these bioconjugates would have been ineffective against F98 wildtype tumors (F98_WT_), which do not express amplified EGFR. If similar studies had been carried out in rats bearing composite tumors consisting of F98_EGFR_ and F98_WT_, we would predict only a modest increase in MST.

Finally, as recently reported by Sun et al. [81], it is noteworthy that a MoAb directed against the stem cell marker CD133, which frequently is expressed on glioma cells, could be used to deliver a heavily boronated dendrimer to specifically target this cell population, both in vitro and in vivo. A significantly longer survival time was seen in BALB/c mice bearing intracerebral CD133 + SU2 glioma cells compared to that of CD133 − SU2 cells. These results suggest that further studies using CD133 targeting, boron containing bioconjugates are warranted to evaluate their potential.

**Fig. 4:**
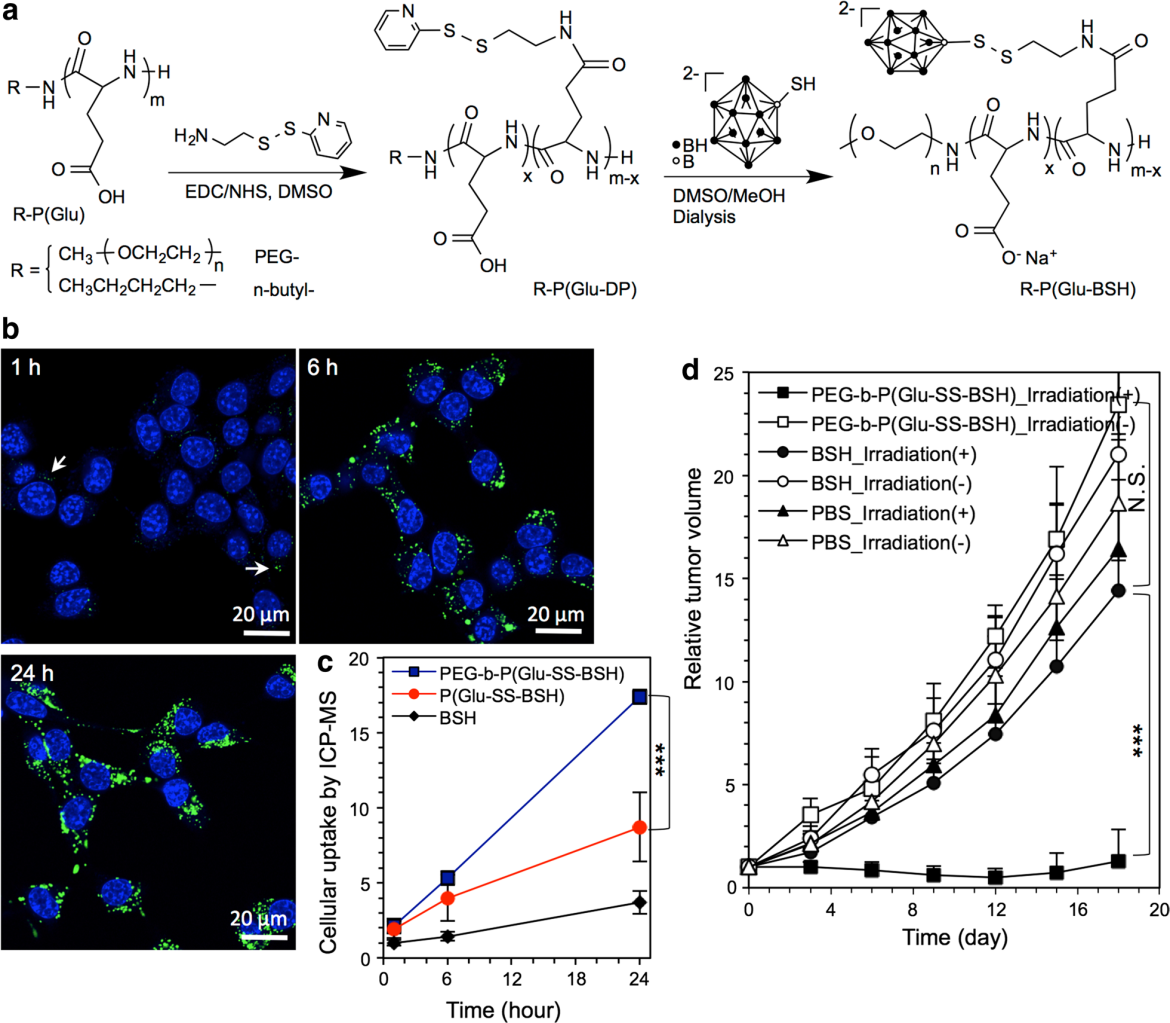
BSH-polymer conjugates for tumor BNCT. **a** Synthetic scheme of BSH-polymer conjugates [PEG-*b*-P(Glu-SS-BSH) and P(Glu-SS-BSH)]; **b** Time-lapsed cellular uptake of PEG-*b*-P(Glu-SS-BSH) by C26 cancer cells was investigated by confocal laser scanning microscopy (CLSM). Both PEG-*b*-P(Glu-SS-BSH) and P(Glu-SS-BSH) were labeled with Alexa488 (green color), and their dose was 20 µg/mL on a BSH basis, while the nuclei were stained with Hoechst (blue color). **c** Relative cellular uptake of BSH, PEG-*b*-P(Glu-SS-BSH) and P(Glu-SS-BSH) was measured by inductively coupled plasma mass spectrometry (ICP-MS). The C26 cancer cells were exposed to BSH, PEG-*b*-P(Glu-SS-BSH) and P(Glu-SS-BSH) for 1, 6 and 24 h (n = 3), at a dose of 100 µg/mL on a BSH basis, while the results were measured by ICP-MS and normalized by comparing with the cellular uptake of BSH at 1 h. The data are expressed as the mean ± SD, ***P < 0.001. **d** Tumor growth ratio of C26 subcutaneous tumors in BALB/c mice that were irradiated with thermal neutrons (1.6–2.2 × 10^12^ neutron/cm^2^) at Kyoto University Reactor (KUR) for 1 h after intravenous injection of phosphate buffered saline (PBS), BSH, and BSH-polymer conjugates for 24 h at a dose of 100 mg/kg on a BSH basis. Reproduced with permission. Copyright 2017, Elsevier [86]

Polymers are alternative carriers for boron compounds, and linkage to them could improve the solubility and pharmacokinetics of these compounds by increasing their circulation half-life and tumor accumulation [82]. BPA is a hydrophobic boron compound, whose cellular uptake is dependent upon the l-amino acid transporter system [83], and conjugation to polymers might also increase its solubility as had complexation with fructose [33]. For example, boronated cationic copolymers, composed of different ratios of acrylamide, N-acryloyl-3-aminophenylboronic acid and N-acryloyl-diaminoethane (the cationic moiety), have been synthesized as delivery agents for boronic acids (Fig. 4) [84]. The molecular weight of the resulting tri-block polymer ranged from 9.98 to 10.21 kDa, which resulted in 14–21 µg/g of boron per gram tumor with an increased cationic monomer ratio in tumor versus normal peri-colonic tissue following intravenous injection of boronic polymers. However, cationic polymers can trigger serious side effects in vivo, such as the induction of cell necrosis via impairment of Na^+^/K^+^-ATPase, thereby resulting in an inflammatory response [85]. Therefore, some polyanionic polymers have been evaluated, such as PEGylated-polyglutamic acid, which has been synthesized by conjugating BSH via a disulfide bond [86]. BSH is hydrophilic and has a higher boron content than BPA, but lower tumor uptake and retention due to its negative charge and low molecular weight. Cellular uptake was significantly improved by conjugating BSH with PEGylated-polyglutamic acid (PEG-*b*-P(Glu-BSH)), which increased tumor cell uptake within 1 h and resulted in a five-fold increase in the tumor boron concentration compared to that of BSH at 24 h [86, 87]. PEG-*b*-P(Glu-BSH), was administered intravenously to BALB/c mice bearing subcutaneous implants of the Colon-26 (C26) carcinoma cell line. This resulted in 70–90 µg of B^10^ per g tumor after a single intravenous injection at the dose of 50 mg/kg with a tumor:blood ratio of 20:1. In vivo BNCT was carried out 24 h after intravenous injection of PEG-*b*-P(Glu-BSH) to tumor-bearing mice, indicating enough ^10^B was delivered to eradicate the tumor. Based on these studies it was concluded that Glu-BSH appeared to be superior to BSH, as evidenced by increased tumor:normal tissue ratios and an improved tumor:blood ratio. However, high uptake in non-target organs [88] and questions relating to their ability to traverse the BBB must be evaluated before biodistribution studies in larger animals are initiated. Recently, functionalized dodecaborate has been linked to albumin and, following intravenous administration, it was effective in achieving tumor targeting and enhanced efficacy against subcutaneous implants of the murine C26 colon carcinoma [89]. This suggested that it might be useful as a delivery agent for extracranial tumors such as head and neck cancer and melanomas.

**Fig. 5:**
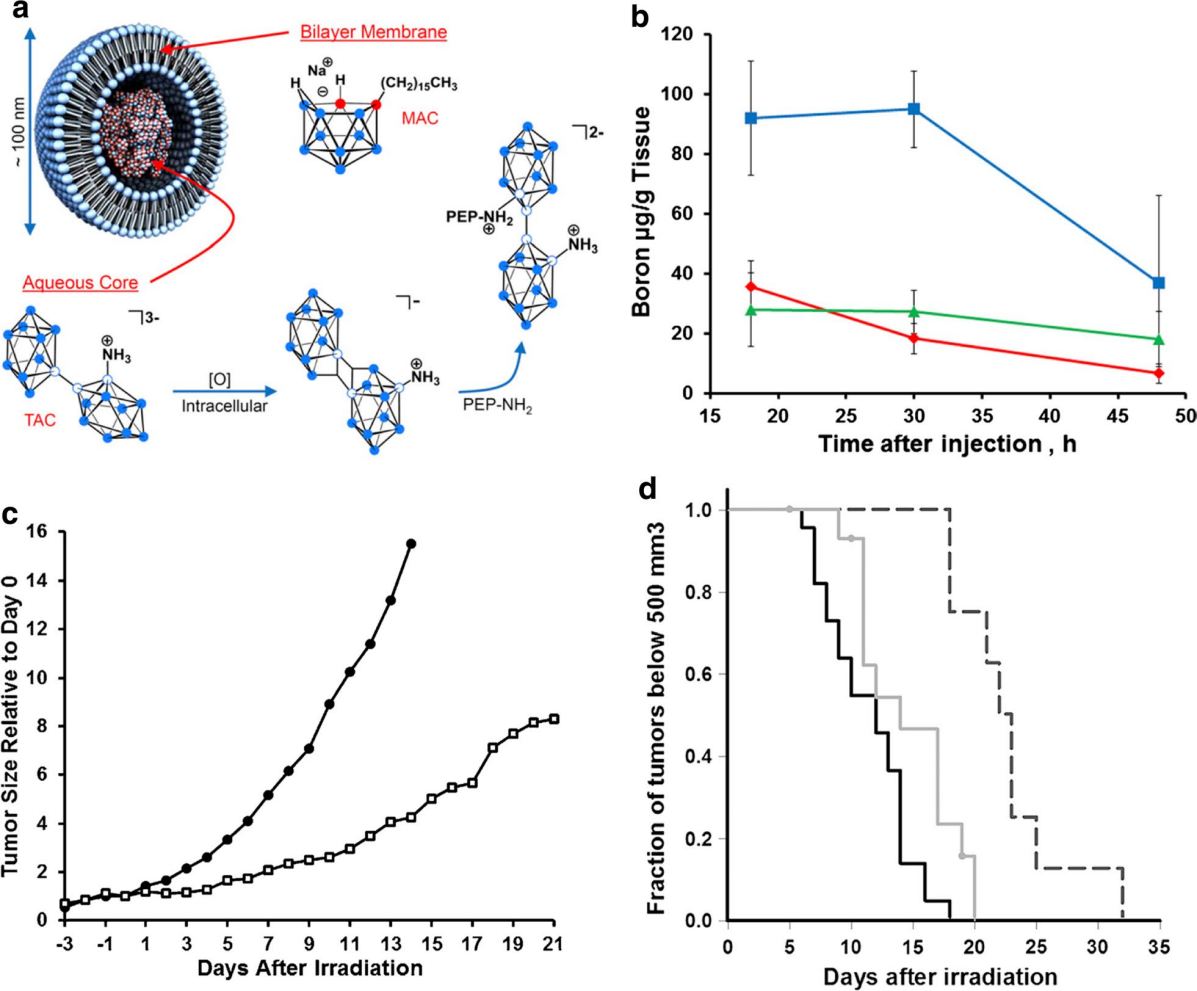
Boron cluster-loaded liposomes for tumor BNCT. **a** Schematic illustration of liposomes incorporating Na_3_ [1-(2′-B_10_H_9_)-2-NH_3_B_10_H_8_] for BNCT. **b** The biodistribution of boron in EMT6 tumor-bearing mice after a single intravenous injection (340–345 µg of boron; red diamond = blood, green triangle = tumor, blue square = liver). **c** Tumor growth curves normalized with respect to mean volume on day 0 after BNCT treatment consisted of a 30-min irradiation following double injection of liposomal suspension (set as the time of irradiation): black circle control group; white square, BNCT group. **d** Kaplan–Meier time-to-event curves indicating time required to reach a 500-mm^3^ tumor volume (solid black line, control group; solid gray line, neutron irradiation-only group; dashed line, BNCT group). Reproduced with permission. Copyright 2013, National Academy of Science [99]

Liposomes, which are vesicles containing an aqueous volume entirely enclosed by a lipid bilayer [90], have been extensively studied, for more than 35 years as potential boron delivery agents [86, 91–98]. The compound Na_3_[1–(2′-B_10_H_9_)-2-NH_3_B_10_H_8_] has been incorporated into the core of liposomes (Fig. 5) and this subsequently was followed by two in vivo studies in mice bearing the EMT6 mammary tumor. The tumor boron concentration in the latter was ~ 40 μg/g at 54 h after a single intravenous injection, after which it gradually decreased [98, 99]. In both studies [97, 98], following BNCT there was slower tumor growth compared to that of the control groups. Boron compounds such as these also can be conjugated to lipids to form boron-loaded liposomes with boron concentrations of 150 ppm. Their in vitro tumoricidal effects also have been demonstrated following neutron irradiation [97].

Targeting moieties such as MoAbs [92], antibodies directed against carcinoembryonic antigens (CEA) [100], transferrin [101], and EGFR [102] also have been introduced on the surface of liposomes to specifically target tumor cells. These immunoliposomes could deliver low molecular weight hydrophobic agents such as BSH that have been incorporated into their lipid bilayers [102, 103], and liposomes can transport large numbers of boron-containing molecules intracellularly, resulting in high tumor boron uptake [104]. Liposomes also have been extensively investigated as delivery agents for a variety of polyhedral boron anions and these studies are described in detail elsewhere [105]. High tumor boron concentrations were attained in vitro when polyhedral boron anions were encapsulated in tumor-selective unilamellar liposomes, and their in vivo therapeutic efficacy has been demonstrated in EMT6 tumor bearing mice [93]. Linkage of boron-containing liposomes to the MoAb cetuximab (C225 or Erbitux™) resulted in specific in vitro molecular targeting of EGFR expressing F98_EGFR_ glioma cells [102]. Boron-containing lipids bearing covalently-bound boron clusters also have been described [105, 106]. These nanoparticles showed no leakage of the encapsulated boron compounds and had the capability of delivering high tumor payloads of boron in mice bearing subcutaneous gliomas and increased survival times following BNCT [106, 107]. However, their large size and high molecular weight would preclude their passage across the BBB in rodents bearing intracranial tumors unless there was disruption of the BBB. This could be accomplished by such methods as the intra-carotid infusion of a hyperosmotic solution of mannitol [49–52], focused ultrasound [108, 109], or direct intratumoral administration by means of CED [64, 110]. Despite all of their potential advantages, boron containing liposomes have yet to be evaluated in animals other than rodents, and their clinical use as boron delivery agents is still to be determined [86, 111].

**Fig. 6:**
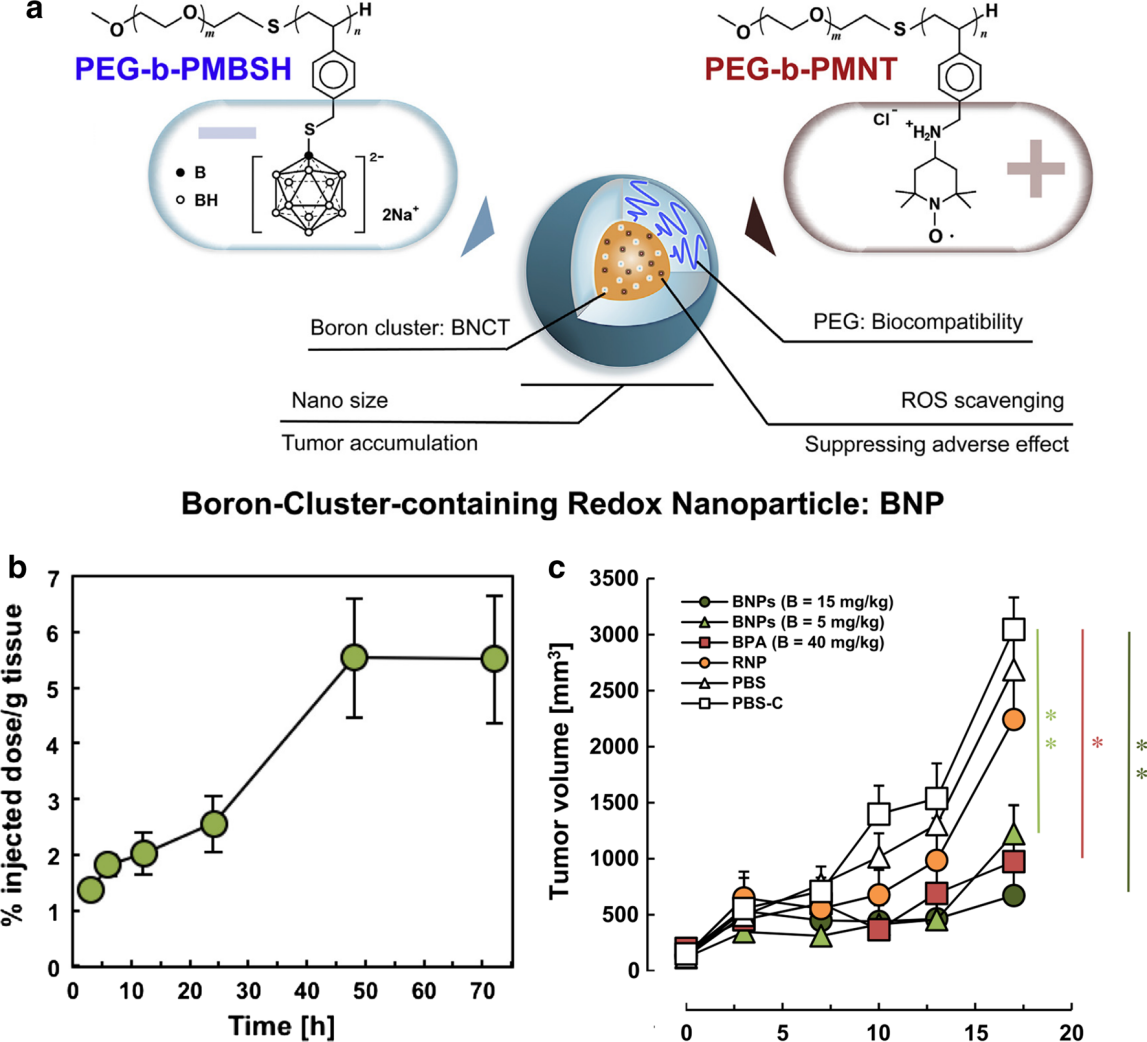
Boron cluster containing redox nanoparticles (BNP) for tumor BNCT. **a** Scheme for preparing boron cluster containing redox nanoparticles. **b** Biodistribution of BNP in tumor-bearing mice. **c** Tumor growth curves of tumor-bearing mice after 40-min thermal neutron irradiation (1.3–1.7 × 10^12^ neutron/cm^2^). Mice with a mean original tumor volume of 140 mm^3^ received BNP at doses of 15 and 5 mg ^10^B/kg. Mice administered BPA–fructose complex at a dose of 40 mg ^10^B/kg were used as the positive control. Mice administered boron cluster containing redox nanoparticles with the same amount of nitroxide radical as in the BNP-treated group at a dose of 15 mg ^10^B/kg and PBS with and without (PBS-C) irradiation were used as negative controls (n = 3, mean ± SD, *P < 0.01, **P < 0.005, Student’s t test). Reproduced with permission. Copyright 2016, Elsevier [117]

Polymeric nanoparticles have been evaluated for drug delivery to metastatic tumors [112] and as potential delivery agents for gadolinium neutron capture therapy (Gd-NCT) [113–115]. Boron-containing micelles were shown to have improved stability, blood circulation time, and tumor accumulation [116]. Recently, boron clusters containing redox nanoparticles have been developed, which have reactive oxygen species scavenging ability, high therapeutic efficacy and minimal side effects (Fig. 6) [117]. They were formed by static interaction of the positively charged BSH-conjugated polymers with the positively charged polymers with redox-responsive groups. These nanoparticles had an extended circulation time in blood and increased uptake in C26 tumors with over 5% of the injected dose per gram tumor at 48 h. They effectively suppressed the tumor growth following BNCT when administered at a dose of 15 mg/kg. In addition, these micelles also could be decorated with folic acid on their surface to increase tumor-specific targeting [118, 119] and achieve higher intracellular boron concentrations [120].

Ending on a positive note, the single most practical major advance in the development of boron delivery agents has been described by Kabalka et al. [121, 122] and Imahori et al. [123, 124]. They have labeled BPA with fluorine-18 for positron emission tomography (PET) in order to determine the tumor uptake of BPA and thereby improve treatment planning [124]. It should be pointed out, however, that PET is usually performed prior to surgical resection of the primary tumor in the case of high grade gliomas, and therefore the imaging data may not reflect the uptake of ^18^F-BPA by residual or recurrent tumor that would be treated by means of BNCT. Nevertheless, ^18^F-BPA PET at least provides some data on the macroscopic uptake of BPA but not on the cellular uptake by individual or clusters of tumor cells, which are too small to be identified by any real time imaging techniques. At present, cellular and subcellular localization of boron can be determined by means of secondary ion mass spectrometry [125–127] or alpha track autoradiography [128] which would allow more accurate dosimetry, but unfortunately these techniques cannot be carried out in real time. Finally, boron compounds also have been conjugated to diethylene-triamine-penta-acetic acid gadolinium (III) dihydrogen (Gd-DTPA) to form a potential theranostic system (Gd/B-NPs) with β-cyclodextrin [129] for tumor localization by MRI and the determination of boron concentrations [130].

## Conclusions

Why has it been so difficult to develop new boron delivery agents for BNCT? Clearly, it has not been for a lack of trying, as evidenced by the voluminous literature beginning in the 1970s on their design and synthesis, as summarized in a number of reviews [21–24]. However, there are still only two drugs in clinical use, BSH and BPA. Objectively, the challenges are much more difficult than the design of chemotherapeutic or tumor imaging agents. Boron delivery agents must not only have tumor selectivity but also deliver amounts far in excess of that required for radiopharmaceuticals to detect tumors by radiodiagnostic modalities such as single photon emission computerized tomography and PET. In contrast to radiopharmaceuticals, these agents must deliver enough ^10^B, presumptively to all tumor cells, in amounts sufficient to sustain a lethal ^10^B(n,α) Li capture reaction (~ 20–50 µg per g tumor or ~ 10^9^ atoms per tumor cell). Furthermore, they must persist in these tumor cells for a sufficient amount of time, and simultaneously clear from surrounding normal tissues to ideally attain a tumor:normal tissue ratio of 3–4:1.

Translating experimental animal data into a clinical biodistribution study represents a significant hurdle that must be overcome. *First*, and most importantly, as of the time of writing there has been a lack of convincing experimental animal data that would warrant the initiation of expensive clinical biodistribution studies for any of the boron delivery agents that we have described in this review. *Second*, there is a major challenge in going from laboratory synthesis to scale up synthesis in a Good Manufacturing Practices (GMP) facility before clinical studies can be initiated. *Third*, these biodistribution studies would have no direct benefit to the patients participating in them other than the altruistic reason that might help other future patients with malignancies that would be treated by means of BNCT. *Fourth*, the issue of funding of such a Phase I clinical biodistribution studies represents a significant hurdle, at least in the United States, where at this time there is very little chance of getting funding from the government or the pharmaceutical industry and where an Investigative New Drug application would require very convincing experimental animal data, including toxicologic evaluation in at least one non-rodent animal species.

What, then, is the best course of action at the present time? *First* and foremost would be to optimize the dosing paradigms for BSH and BPA. Clinical data generated by the Swedish group [38, 39, 131] suggest that increasing the dose of BPA and the infusion time resulted in improved survival in patients with high grade gliomas who had been treated with BNCT. *Second*, methods should be explored to enhance the delivery of BSH and BPA, both in brain tumor patients and patients that have had recurrent tumors of the head and neck region. Two of us (Barth and Yang) have convincingly demonstrated that transient disruption of the BBB by intracarotid infusion of a hyperosmotic solution of mannitol, combined with administration of either BSH or BPA, resulted in a threefold increase in tumor boron concentrations in F98 glioma bearing rats [49–52]. This enhanced tumor uptake of BSH and BPA resulted in a three- to four-fold increase in MST following BNCT. Although this procedure has been used clinically to administer cytoreductive chemotherapeutic agents to patients with high grade gliomas, it requires a very specialized team, which may make it difficult to carry out in patients who will be receiving BNCT [132–134]. An alternative approach [135–137] could be the use of pulsed ultrasound [109, 138] initially to enhance tumor uptake of ^18^F–BPA for PET imaging. ^18^F–BPA PET imaging [121–124] is now a well-established technique used as part of the treatment planning protocols both in Japan and Finland, the two countries where the largest number of patients have been treated by BNCT. Although some of the clinical results that have been obtained in these two countries have been impressive [44], especially in the treatment of genital cancers [18]. It remains to be determined if the results would be sufficient to convince a broader group of physicians, who are taking care of cancer patients on a day-to-day basis, that BNCT would be worth pursuing The challenge to those of us who have been working in this field is to come up with truly convincing data!

## Abbreviations

BBB: blood brain barrier
BNCT: boron neutron capture therapy
BPA: boronophenylalanine
BPA-F: boronophenylalanine-fructose complex
BSH: sodium borocaptate
CEA: carcinoembryonic antigen
CED: convection enhanced delivery
EGFR: epidermal growth factor receptor
EGFR_vIII_: EGFR variant
LET: linear energy transfer
MoAb: monoclonal antibody
MST: mean survival time
PEG: polyethylene glycol
PET: positron emission tomography
ROS: reactive oxygen species
SIMS: secondary ion mass spectrometry
TK1: thymidine kinase 1
VEGF: vascular endothelial growth factor
VEGFR: vascular endothelial growth factor receptor

## References

[1] Barth RF, Coderre JA, Vicente MG, Blue TE (2005). Boron neutron capture therapy of cancer: current status and future prospects. Clin Cancer Res.

[2] Hatanaka H, Karin ABMF, Laws E (1991). Boron neutron capture therapy for brain tumors. Glioma.

[3] Nakagawa Y, Pooh K, Kobayashi T, Kageji T, Uyama S, Matsumura A (2003). Clinical review of the Japanese experience with boron neutron capture therapy and a proposed strategy using epithermal neutron beams. J Neurooncol.

[4] Miyatake S, Kawabata S, Kajimoto Y, Aoki A, Yokoyama K, Yamada M (2005). Modified boron neutron capture therapy for malignant gliomas performed using epithermal neutron and two boron compounds with different accumulation mechanisms: an efficacy study based on findings on neuroimages. J Neurosurg.

[5] Miyatake S, Kawabata S, Yokoyama K, Kuroiwa T, Michiue H, Sakurai Y (2009). Survival benefit of boron neutron capture therapy for recurrent malignant gliomas. J Neurooncol.

[6] Kankaanranta L, Saarilahti K, Makitie A, Valimaki P, Tenhunen M, Joensuu H (2011). Boron neutron capture therapy (BNCT) followed by intensity modulated chemoradiotherapy as primary treatment of large head and neck cancer with intracranial involvement. Radiother Oncol.

[7] Kankaanranta L, Seppala T, Koivunoro H, Saarilahti K, Atula T, Collan J (2012). Boron neutron capture therapy in the treatment of locally recurred head-and-neck cancer: final analysis of a phase I/II trial. Int J Radiat Oncol Biol Phys.

[8] Ariyoshi Y, Miyatake S, Kimura Y, Shimahara T, Kawabata S, Nagata K (2007). Boron neuron capture therapy using epithermal neutrons for recurrent cancer in the oral cavity and cervical lymph node metastasis. Oncol Rep.

[9] Kimura Y, Ariyoshi Y, Miyatake S, Shimahara M, Kawabata S, Ono K (2009). Boron neutron capture therapy for papillary cystadenocarcinoma in the upper lip: a case report. Int J Oral Maxillofac Surg.

[10] Kimura Y, Ariyoshi Y, Shimahara M, Miyatake S, Kawabata S, Ono K (2009). Boron neutron capture therapy for recurrent oral cancer and metastasis of cervical lymph node. Appl Radiat Isot.

[11] Aihara T, Hiratsuka J, Morita N, Uno M, Sakurai Y, Maruhashi A (2006). First clinical case of boron neutron capture therapy for head and neck malignancies using 18F-BPA PET. Head Neck.

[12] Kato I, Ono K, Sakurai Y, Ohmae M, Maruhashi A, Imahori Y (2004). Effectiveness of BNCT for recurrent head and neck malignancies. Appl Radiat Isot.

[13] Kato I, Fujita Y, Maruhashi A, Kumada H, Ohmae M, Kirihata M (2009). Effectiveness of boron neutron capture therapy for recurrent head and neck malignancies. Appl Radiat Isot.

[14] Mishima Y, Honda C, Ichihashi M, Obara H, Hiratsuka J, Fukuda H (1989). Treatment of malignant melanoma by single thermal neutron capture therapy with melanoma-seeking 10B-compound. Lancet.

[15] Mishima Y, Mishima Y (1996). Selective thermal neutron capture therapy of cancer cells using their specific metabolic activities—melanoma as prototype. Cancer neutron capture therapy.

[16] Yong Z, Song Z, Zhou Y, Liu T, Zhang Z, Zhao Y (2016). Boron neutron capture therapy for malignant melanoma: first clinical case report in China. Chin J Cancer Res.

[17] Hiratsuka JF, Sauerwein WAGEA (2012). Malignant melanoma. Neutron capture therapy.

[18] Hiratsuka J, Kamitani N, Tanaka R, et al. Boron neutron capture therapy for vulvar melanoma and extramammary Paget’s disease of the genital regions with curative clinical responses. Chin J Cancer. 2018, **(In press)**. 10.1186/s40880-018-0297-9 PMC6006671 29914570

[19] Mitsumoto TY, Yajima S, Tsutsui H. Cyclotron-based neutron source for BNCT. New challenges in neutron capture therapy 2010: proceedings of the 14th international congress on neutron capture therapy. 2013. p. 519-22.

[20] Smick T. A compact neutron source designed for the hospital environment. https://www.neutrontherapeutics.com/technology/. 2017. Accessed 3-19-2018.

[21] Sibrian-Vazquez M, Vicente MGH, Hosmane NS (2011). Boron tumor-delivery for BNCT: Recent developments and perspectives. Boron science: new technologies and applications.

[22] Nakamura H, Kirihata M, Sauerwein WAG, Wittig A, Moss R, Nakagawa Y (2012). Boron compounds: new candidates for boron carriers in BNCT. Neutron capture therapy.

[23] Soloway AH, Tjarks W, Barnum BA, Rong FG, Barth RF, Codogni IM (1998). The chemistry of neutron capture therapy. Chem Rev.

[24] Hawthorne MF, Lee MW (2003). A critical assessment of boron target compounds for boron neutron capture therapy. J Neurooncol.

[25] Miller HC, Miller NE, Muetterties EL (1964). Chemistry of boranes. XX. Syntheses of polyhedral boranes. Inorg Chem.

[26] Soloway AH, Hatanaka H, Davis MA (1967). Penetration of brain and brain tumor. VII. Tumor-binding sulfhydryl boron compounds. J Med Chem.

[27] Hatanaka H, Nakagawa Y (1994). Clinical results of long-surviving brain tumor patients who underwent boron neutron capture therapy. Int J Radiat Oncol Biol Phys.

[28] Wittig AH, Hideghety K, Paquis P, Heimans J, Sauerwein W, Moss RL, Wittig A (2002). Current clinical results of the EORTC-study 11961. Research and development in neutron capture therapy.

[29] Vos MJ, Turowski B, Zanella FE, Paquis P, Siefert A, Hideghety K (2005). Radiologic findings in patients treated with boron neutron capture therapy for glioblastoma multiforme within EORTC trial 11961. Int J Radiat Oncol Biol Phys.

[30] Snyder HR, Reedy AJ, Lennarz WJ (1958). Synthesis of aromatic boronic acids. Aldehydo boronic acids and a boronic acid analog of tyrosine. J Am Chem Soc.

[31] Mishima Y, Larsson B, Crawford J, Weinreich R (1997). Melanoma and nonmelanoma neutron capture therapy using gene therapy: overview. Advances in neutron capture therapy.

[32] Coderre JA, Glass JD, Fairchild RG, Micca PL, Fand I, Joel DD (1990). Selective delivery of boron by the melanin precursor analogue p-boronophenylalanine to tumors other than melanoma. Can Res.

[33] Yoshino K, Suzuki A, Mori Y, Kakihana H, Honda C, Mishima Y (1989). Improvement of solubility of p-boronophenylalanine by complex formation with monosaccharides. Strahlenther Onkol.

[34] Chadha M, Capala J, Coderre JA, Elowitz EH, Iwai J, Joel DD (1998). Boron neutron-capture therapy (BNCT) for glioblastoma multiforme (GBM) using the epithermal neutron beam at the Brookhaven National Laboratory. Int J Radiat Oncol Biol Phys.

[35] Diaz AZ (2003). Assessment of the results from the phase I/II boron neutron capture therapy trials at the Brookhaven National Laboratory from a clinician’s point of view. J Neurooncol.

[36] Kankaanranta L, Seppälä T, Koivunoro H, Valimaki P, Beule A, Collan J, Kortesniemi M, Uusi-Simola J, Kotiluoto P, Auterinen I, et al. BPA-based BNCT in the treatment of glioblastoma multiforme: a dose escalation study. In: Zonta AA, Roveda L, Barth RF, editors. 13th international congress on neutron capture therapy, a new option against cancer. vol 30, 2008.

[37] Kankaanranta L, Seppala T, Koivunoro H, Valimaki P, Beule A, Collan J (2011). L-boronophenylalanine-mediated boron neutron capture therapy for malignant glioma progressing after external beam radiation therapy: a phase I study. Int J Radiat Oncol Biol Phys.

[38] Sköld K, Stenstam B, Diaz AZ, Giusti V, Pellettieri L, Hopewell JW (2010). Boron neutron capture therapy for glioblastoma multiforme: advantage of prolonged infusion of BPA-f. Acta Neurol Scand.

[39] Hopewell JW, Gorlia T, Pellettieri L, Giusti V, H-Stenstam B, Skold K (2011). Boron neutron capture therapy for newly diagnosed glioblastoma multiforme: an assessment of clinical potential. Appl Radiat Isot.

[40] Kageji T, Mizobuchi Y, Nagahiro S, Nakagawa Y, Kumada H (2011). Long-survivors of glioblastoma treated with boron neutron capture therapy (BNCT). Appl Radiat Isot.

[41] Kawabata S, Miyatake S, Kuroiwa T, Yokoyama K, Doi A, Iida K (2009). Boron neutron capture therapy for newly diagnosed glioblastoma. J Radiat Res..

[42] Miyatake S, Tamura Y, Kawabata S, Iida K, Kuroiwa T, Ono K (2007). Boron neutron capture therapy for malignant tumors related to meningiomas. Neurosurgery.

[43] Suzuki M, Sakurai Y, Nagata K, Kinashi Y, Masunaga S, Ono K (2006). Impact of intra-arterial administration of boron compounds on dose-volume histograms in boron neutron capture therapy for recurrent head-and-neck tumors. Int J Radiat Oncol Biol Phys.

[44] Barth RF, Vicente MH, Harling OK, Kiger W, Riley KJ, Binns PJ (2012). Current status of boron neutron capture therapy of high grade gliomas and recurrent head and neck cancer. Radiat Oncol.

[45] Moss RL (2014). Critical review, with an optimistic outlook, on boron neutron capture therapy (BNCT). Appl Radiat Isot.

[46] Goodman JH, Yang W, Barth RF, Gao Z, Boesel CP, Staubus AE (2000). Boron neutron capture therapy of brain tumors: biodistribution, pharmacokinetics, and radiation dosimetry sodium borocaptate in patients with gliomas. Neurosurgery.

[47] Koivunoro H, Hippelainen E, Auterinen I, Kankaanranta L, Kulvik M, Laakso J (2015). Biokinetic analysis of tissue boron (^10^B) concentrations of glioma patients treated with BNCT in Finland. Appl Radiat Isot.

[48] Chen R, Smith-Cohn M, Cohen AL, Colman H (2017). Glioma subclassifications and their clinical significance. Neurotherapeutics.

[49] Yang W, Barth RF, Carpenter DE, Moeschberger ML, Goodman JH (1996). Enhanced delivery of boronophenylalanine for neutron capture therapy by means of intracarotid injection and blood-brain barrier disruption. Neurosurgery.

[50] Yang W, Barth RF, Rotaru JH, Moeschberger ML, Joel DD, Nawrocky MM (1997). Enhanced survival of glioma bearing rats following boron neutron capture therapy with blood-brain barrier disruption and intracarotid injection of boronophenylalanine. J Neurooncol.

[51] Yang W, Barth RF, Rotaru JH, Moeschberger ML, Joel DD, Nawrocky MM (1997). Boron neutron capture therapy of brain tumors: enhanced survival following intracarotid injection of sodium borocaptate with or without blood-brain barrier disruption. Int J Radiat Oncol Biol Phys.

[52] Barth RF, Yang W, Rotaru JH, Moeschberger ML, Joel DD, Nawrocky MM (1997). Boron neutron capture therapy of brain tumors: enhanced survival following intracarotid injection of either sodium borocaptate or boronophenylalanine with or without blood-brain barrier disruption. Can Res.

[53] Barth RF, Matalka KZ, Bailey MQ, Staubus AE, Soloway AH, Moeschberger ML (1994). A nude rat model for neutron capture therapy of human intracerebral melanoma. Int J Radiat Oncol Biol Phys.

[54] Tajes M, Ramos-Fernandez E, Weng-Jiang X, Bosch-Morato M, Guivernau B, Eraso-Pichot A (2014). The blood-brain barrier: structure, function and therapeutic approaches to cross it. Mol Membr Biol.

[55] Daneman R, Prat A (2015). The blood-brain barrier. Cold Spring Harbor Perspect Biol.

[56] Semioshkin A, Nizhnik E, Godovikov I, Starikova Z, Bregadze V (2007). Reactions of oxonium derivatives of [B12H12]2 − with amines: synthesis and structure of novel B12-based ammonium salts and amino acids. J Organomet Chem.

[57] Kabalka GW, Wu Z, Yao M-L (2008). Synthesis of a series of boronated unnatural cyclic amino acids as potential boron neutron capture therapy agents. Appl Organomet Chem.

[58] Kabalka GW, Yao ML, Marepally SR, Chandra S (2009). Biological evaluation of boronated unnatural amino acids as new boron carriers. Appl Radiat Isot.

[59] Kabalka GW, Shaikh AL, Barth RF, Huo T, Yang W, et al. Boronated unnatural amino acids as new boron carriers for BNCT. In: Liberman S, Kreiner A, Casal M, Menendez P, Schwint A, et al., editor. New Challenges in Neutron Capture Therapy 2010: proceedings of the 14th international congress on neutron capture therapy. Comisión Nacional de Energía Atómica; October 25–29, 2010, Buenos Aires, Argentina, p. 364–7.

[60] Kabalka GW, Shaikh AL, Barth RF, Huo T, Yang W, Gordnier PM (2011). Boronated unnatural cyclic amino acids as potential delivery agents for neutron capture therapy. Appl Radiat Isot.

[61] Barth RF, Kabalka GW, Yang W, Huo T, Nakkula RJ, Shaikh AL (2014). Evaluation of unnatural cyclic amino acids as boron delivery agents for treatment of melanomas and gliomas. Appl Radiat Isot.

[62] Mier W, Gabel D, Haberkorn U (2004). Conjugation of the closo-borane mereaptoundeca-hydrododecaborate (BSH) to a tumor selective peptide. Anorg Allg Chem.

[63] Backer MV, Gaynutdinov TI, Patel V, Bandyopadhyaya AK, Thirumamagal BT, Tjarks W, Barth RF (2005). Vascular endothelial growth factor selectively targets boronated dendrimers to tumor vasculature. Mol Cancer Ther.

[64] Yang W, Barth RF, Wu G, Kawabata S, Sferra TJ, Bandyopadhyaya AK (2006). Molecular targeting and treatment of EGFRvIII-positive gliomas using boronated monoclonal antibody L8A4. Clin Cancer Res.

[65] Wu G, Yang W, Barth RF, Kawabata S, Swindall M, Bandyopadhyaya AK (2007). Molecular targeting and treatment of an epidermal growth factor receptor-positive glioma using boronated cetuximab. Clin Cancer Res.

[66] Yang W, Wu G, Barth RF, Swindall MR, Bandyopadhyaya AK, Tjarks W (2008). Molecular targeting and treatment of composite EGFR and EGFRvIII-positive gliomas using boronated monoclonal antibodies. Clin Cancer Res.

[67] Al-Madhoun AS, Johnsamuel J, Barth RF, Tjarks W, Eriksson S (2004). Evaluation of human thymidine kinase 1 substrates as new candidates for boron neutron capture therapy. Can Res.

[68] Barth RF, Yang W, Wu G, Swindall M, Byun Y, Narayanasamy S (2008). Thymidine kinase 1 as a molecular target for boron neutron capture therapy of brain tumors. Proc Natl Acad Sci USA.

[69] Sjuvarsson E, Damaraju VL, Mowles D, Sawyer MB, Tiwari R, Agarwal HK (2013). Cellular influx, efflux, and anabolism of 3-carboranyl thymidine analogs: potential boron delivery agents for neutron capture therapy. J Pharmacol Exp Ther.

[70] Lewis O, Woolley M, Johnson D, Rosser A, Barua NU, Bienemann AS (2016). Chronic, intermittent convection-enhanced delivery devices. J Neurosci Methods.

[71] Kawabata S, Yang W, Barth RF, Wu G, Huo T, Binns PJ (2011). Convection enhanced delivery of carboranylporphyrins for neutron capture therapy of brain tumors. J Neurooncol.

[72] Barth RF, Yang W, Nakkula RJ, Byun Y, Tjarks W, Wu LC (2015). Evaluation of TK1 targeting carboranyl thymidine analogs as potential delivery agents for neutron capture therapy of brain tumors. Appl Radiat Isot.

[73] Renner MW, Miura M, Easson MW, Vicente MG (2006). Recent progress in the syntheses and biological evaluation of boronated porphyrins for boron neutron-capture therapy. Anticancer Agents Med Chem.

[74] Ol’shevskaya VA, Zaytsev AV, Savchenko AN (2007). Boronated porphyrins and chlorins as potential anticancer drugs. Bull Korean Chem Soc.

[75] Kahl S, Koo M: Synthesis and properties of tebrakis-carborane-carboxylate esters of 2;4-bis (−dehydroryethyl) dereteroporphyrin IX. In: Allen B, Moore D, Harrington B, editor. Progress in neutron capture therapy. New York: Plenium Press; 1992. p. 223–6.

[76] Crossley EL, Ziolkowski EJ, Coderre JA, Rendina LM (2007). Boronated DNA-binding compounds as potential agents for boron neutron capture therapy. Mini Rev Med Chem.

[77] Orlova AV, Kononov LO, Kimel BG, Sivaev IB, Bregadze VI (2006). Conjugates of polyhedral boron compounds with carbohydrates. 4. hydrolytic stability of carborane–lactose conjugates depends on the structure of a spacer between the carborane cage and sugar moiety. Appl Organomet Chem.

[78] Capala J, Barth RF, Bendayan M, Lauzon M, Adams DM, Soloway AH (1996). Boronated epidermal growth factor as a potential targeting agent for boron neutron capture therapy of brain tumors. Bioconjug Chem.

[79] Yang W, Barth RF, Wu G, Huo T, Tjarks W, Ciesielski M (2009). Convection enhanced delivery of boronated EGF as a molecular targeting agent for neutron capture therapy of brain tumors. J Neurooncol.

[80] Yang W, Barth RF, Wu G, Tjarks W, Binns P, Riley K (2009). Boron neutron capture therapy of EGFR or EGFRvIII positive gliomas using either boronated monoclonal antibodies or epidermal growth factor as molecular targeting agents. Appl Radiat Isot.

[81] Sun T, Li Y, Huang Y, Zhang Z, Yang W, Du Z (2016). Targeting glioma stem cells enhances anti-tumor effect of boron neutron capture therapy. Oncotarget.

[82] Duncan R, Vicent MJ (2013). Polymer therapeutics-prospects for 21st century: the end of the beginning. Adv Drug Deliv Rev.

[83] Detta A, Cruickshank GS (2009). L-amino acid transporter-1 and boronophenylalanine-based boron neutron capture therapy of human brain tumors. Can Res.

[84] Azab AK, Srebnik M, Doviner V, Rubinstein A (2005). Targeting normal and neoplastic tissues in the rat jejunum and colon with boronated, cationic acrylamide copolymers. J Control Release.

[85] Wei X, Shao B, He Z, Ye T, Luo M, Sang Y (2015). Cationic nanocarriers induce cell necrosis through impairment of Na(+)/K(+)-ATPase and cause subsequent inflammatory response. Cell Res.

[86] Mi P, Yanagie H, Dewi N, Yen HC, Liu X, Suzuki M (2017). Block copolymer-boron cluster conjugate for effective boron neutron capture therapy of solid tumors. J Control Release.

[87] Dewi N, Mi P, Yanagie H, Sakurai Y, Morishita Y, Yanagawa M (2016). In vivo evaluation of neutron capture therapy effectivity using calcium phosphate-based nanoparticles as Gd-DTPA delivery agent. J Cancer Res Clin Oncol.

[88] Luderer MJ, de la Puente P, Azab AK (2015). Advancements in tumor targeting strategies for boron neutron capture therapy. Pharm Res.

[89] Kikuchi S, Kanoh D, Sato S, Sakurai Y, Suzuki M, Nakamura H (2016). Maleimide-functionalized closo-dodecaborate albumin conjugates (MID-AC): unique ligation at cysteine and lysine residues enables efficient boron delivery to tumor for neutron capture therapy. J Control Release.

[90] Yanagie H, Ogata A, Sugiyama H, Eriguchi M, Takamoto S, Takahashi H (2008). Application of drug delivery system to boron neutron capture therapy for cancer. Expert Opin Drug Deliv.

[91] Shelly K, Feakes DA, Hawthorne MF, Schmidt PG, Krisch TA, Bauer WF (1992). Model studies directed toward the boron neutron-capture therapy of cancer: boron delivery to murine tumors with liposomes. Proc Natl Acad Sci USA.

[92] Yanagie H, Tomita T, Kobayashi H, Fujii Y, Takahashi T, Hasumi K (1991). Application of boronated anti-CEA immunoliposome to tumour cell growth inhibition in in vitro boron neutron capture therapy model. Br J Cancer.

[93] Feakes DA, Shelly K, Hawthorne MF (1995). Selective boron delivery to murine tumors by lipophilic species incorporated in the membranes of unilamellar liposomes. Proc Natl Acad Sci USA.

[94] Yanagie H, Tomita T, Kobayashi H, Fujii Y, Nonaka Y, Saegusa Y (1997). Inhibition of human pancreatic cancer growth in nude mice by boron neutron capture therapy. Br J Cancer.

[95] Watson-Clark RA, Banquerigo ML, Shelly K, Hawthorne MF, Brahn E (1998). Model studies directed toward the application of boron neutron capture therapy to rheumatoid arthritis: boron delivery by liposomes in rat collagen-induced arthritis. Proc Natl Acad Sci USA.

[96] Yanagie H, Kobayashi H, Takeda Y, Yoshizaki I, Nonaka Y, Naka S (2002). Inhibition of growth of human breast cancer cells in culture by neutron capture using liposomes containing B-10. Biomed Pharmacother.

[97] Koganei H, Ueno M, Tachikawa S, Tasaki L, Ban HS, Suzuki M (2013). Development of high boron content liposomes and their promising antitumor effect for neutron capture therapy of cancers. Bioconjug Chem.

[98] Tachikawa S, Miyoshi T, Koganei H, El-Zaria ME, Vinas C, Suzuki M (2014). Spermidinium closo-dodecaborate-encapsulating liposomes as efficient boron delivery vehicles for neutron capture therapy. Chem Commun.

[99] Kueffer PJ, Maitz CA, Khan AA, Schuster SA, Shlyakhtina NI, Jalisatgi SS (2013). Boron neutron capture therapy demonstrated in mice bearing EMT6 tumors following selective delivery of boron by rationally designed liposomes. Proc Natl Acad Sci USA.

[100] Yanagie H, Tomita T, Kobayashi H, Fujii Y, Nonaka Y, Saegusa Y (1997). Inhibition of human pancreatic cancer growth in nude mice by boron neutron capture therapy. Br J Cancer.

[101] Maruyama K, Ishida O, Kasaoka S, Takizawa T, Utoguchi N, Shinohara A (2004). Intracellular targeting of sodium mercaptoundecahydrododecaborate (BSH) to solid tumors by transferrin-PEG liposomes, for boron neutron-capture therapy (BNCT). J Control Release.

[102] Pan X, Wu G, Yang W, Barth RF, Tjarks W, Lee RJ (2007). Synthesis of cetuximab-immunoliposomes via a cholesterol-based membrane anchor for targeting of EGFR. Bioconjug Chem.

[103] Nakamura H, Hosmane NS (2012). Liposomal boron delivery system for neutron capture therapy of cancer. Boron science: new technologies and applications.

[104] Altieri S, Balzi M, Bortolussi S, Bruschi P, Ciani L, Clerici AM (2009). Carborane derivatives loaded into liposomes as efficient delivery systems for boron neutron capture therapy. J Med Chem.

[105] Feakes DA, Hosmane NS (2011). Design and development of polyhedral borane anions for liposomal delivery. Boron science: new technologies and applications.

[106] Li T, Hamdi J, Hawthorne MF (2006). Unilamellar liposomes with enhanced boron content. Bioconjug Chem.

[107] Doi A, Kawabata S, Iida K, Yokoyama K, Kajimoto Y, Kuroiwa T (2008). Tumor-specific targeting of sodium borocaptate (BSH) to malignant glioma by transferrin-PEG liposomes: a modality for boron neutron capture therapy. J Neuro Oncol.

[108] Liu HL, Hsu PH, Lin CY, Huang CW, Chai WY, Chu PC (2016). Focused ultrasound enhances central nervous system delivery of bevacizumab for malignant glioma treatment. Radiology.

[109] Burgess A, Shah K, Hough O, Hynynen K (2015). Focused ultrasound-mediated drug delivery through the blood-brain barrier. Expert Rev Neurother.

[110] Yang W, Barth RF, Adams DM, Ciesielski MJ, Fenstermaker RA, Shukla S (2002). Convection-enhanced delivery of boronated epidermal growth factor for molecular targeting of EGF receptor-positive gliomas. Can Res.

[111] Cabral H, Makino J, Matsumoto Y, Mi P, Wu H, Nomoto T (2015). Systemic targeting of lymph node metastasis through the blood vascular system by using size-controlled nanocarriers. ACS Nano.

[112] Kataoka K, Harada A, Nagasaki Y (2001). Block copolymer micelles for drug delivery: design, characterization and biological significance. Adv Drug Deliv Rev.

[113] Yi Y, Lin G, Chen S, Liu J, Zhang H, Mi P (2018). Polyester micelles for drug delivery and cancer theranostics: current achievements, progresses and future perspectives. Mater Sci Eng C..

[114] Mi P, Dewi N, Yanagie H, Kokuryo D, Suzuki M, Sakurai Y (2015). Hybrid calcium phosphate-polymeric micelles incorporating gadolinium chelates for imaging-guided gadolinium neutron capture tumor therapy. ACS Nano.

[115] Dewi N, Yanagie H, Zhu H, Demachi K, Shinohara A, Yokoyama K (2013). Tumor growth suppression by gadolinium-neutron capture therapy using gadolinium-entrapped liposome as gadolinium delivery agent. Biomed Pharmacother.

[116] Sumitani S, Oishi M, Nagasaki Y (2011). Carborane confined nanoparticles for boron neutron capture therapy: improved stability, blood circulation time and tumor accumulation. React Funct Polym.

[117] Gao Z, Horiguchi Y, Nakai K, Matsumura A, Suzuki M, Ono K (2016). Use of boron cluster-containing redox nanoparticles with ROS scavenging ability in boron neutron capture therapy to achieve high therapeutic efficiency and low adverse effects. Biomaterials.

[118] Mandal S, Bakeine GJ, Krol S, Ferrari C, Clerici AM, Zonta C (2011). Design, development and characterization of multi-functionalized gold nanoparticles for biodetection and targeted boron delivery in BNCT applications. Appl Radiat Isot.

[119] Achilli C, Grandi S, Ciana A, Guidetti GF, Malara A, Abbonante V (2014). Biocompatibility of functionalized boron phosphate (BPO4) nanoparticles for boron neutron capture therapy (BNCT) application. Nanomed Nanotechnol Biol Med.

[120] Dai C, Cai F, Hwang KC, Zhou Y, Zhang Z, Liu X (2013). Folate receptor-mediated boron-10 containing carbon nanoparticles as potential delivery vehicles for boron neutron capture therapy of nonfunctional pituitary adenomas. Sci China Life Sci..

[121] Kabalka GW, Smith GT, Dyke JP, Reid WS, Longford CP, Roberts TG (1997). Evaluation of fluorine-18-BPA-fructose for boron neutron capture treatment planning. J Nucl Med.

[122] Kabalka GW, Nichols TL, Smith GT, Miller LF, Khan MK, Busse PM (2003). The use of positron emission tomography to develop boron neutron capture therapy treatment plans for metastatic malignant melanoma. J Neurooncol.

[123] Imahori Y, Ueda S, Ohmori Y, Sakae K, Kusuki T, Kobayashi T (1998). Positron emission tomography-based boron neutron capture therapy using boronophenylalanine for high-grade gliomas: part II. Clin Cancer Res.

[124] Imahori Y, Ueda S, Ohmori Y, Kusuki T, Ono K, Fujii R (1998). Fluorine-18-labeled fluoroboronophenylalanine PET in patients with glioma. J Nucl Med.

[125] Chandra S, Barth RF, Haider SA, Yang W, Huo T, Shaikh AL (2013). Biodistribution and subcellular localization of an unnatural boron-containing amino acid (cis-ABCPC) by imaging secondary ion mass spectrometry for neutron capture therapy of melanomas and gliomas. PLoS ONE.

[126] Chandra S, Ahmad T, Barth RF, Kabalka GW (2014). Quantitative evaluation of boron neutron capture therapy (BNCT) drugs for boron delivery and retention at subcellular-scale resolution in human glioblastoma cells with imaging secondary ion mass spectrometry (SIMS). J Microsc.

[127] Chandra S, Parker DJ, Barth RF, Pannullo SC (2016). Quantitative imaging of magnesium distribution at single-cell resolution in brain tumors and infiltrating tumor cells with secondary ion mass spectrometry (SIMS). J Neurooncol.

[128] Woollard JE, Blue TE, Curran JF, Mengers TF, Barth RF (1990). An alpha autoradiographic technique for determination of 10B concentrations in blood and tissue. Nucl Instrum Methods Phys Res Sect A.

[129] Geninatti-Crich S, Alberti D, Szabo I, Deagostino A, Toppino A, Barge A (2011). MRI-guided neutron capture therapy by use of a dual gadolinium/boron agent targeted at tumour cells through upregulated low-density lipoprotein transporters. Chemistry.

[130] Geninatti-Crich S, Alberti D, Szabo I, Deagostino A, Toppino A, Barge A (2011). MRI-guided neutron capture therapy by use of a dual gadolinium/boron agent targeted at tumour cells through upregulated low-density lipoprotein transporters. Chemistry.

[131] Sköld K, Gorlia T, Pellettieri L, Giusti V (2010). B HS, Hopewell JW. Boron neutron capture therapy for newly diagnosed glioblastoma multiforme: an assessment of clinical potential. Br J Radiol.

[132] Neuwelt EA, Schiff D (2015). Primary CNS lymphoma: a landmark trial and the next steps. Neurology.

[133] Doolittle ND, Muldoon LL, Culp AY, Neuwelt EA (2014). Delivery of chemotherapeutics across the blood-brain barrier: challenges and advances. Adv Pharmacol.

[134] Doolittle ND, Dosa E, Fu R, Muldoon LL, Maron LM, Lubow MA (2013). Preservation of cognitive function in primary CNS lymphoma survivors a median of 12 years after enhanced chemotherapy delivery. J Clin Oncol.

[135] Jahnke K, Kraemer DF, Knight KR, Fortin D, Bell S, Doolittle ND (2008). Intraarterial chemotherapy and osmotic blood-brain barrier disruption for patients with embryonal and germ cell tumors of the central nervous system. Cancer.

[136] Angelov L, Doolittle ND, Kraemer DF, Siegal T, Barnett GH, Peereboom DM (2009). Blood-brain barrier disruption and intra-arterial methotrexate-based therapy for newly diagnosed primary CNS lymphoma: a multi-institutional experience. J Clin Oncol.

[137] Guillaume DJ, Doolittle ND, Gahramanov S, Hedrick NA, Delashaw JB, Neuwelt EA (2010). Intra-arterial chemotherapy with osmotic blood-brain barrier disruption for aggressive oligodendroglial tumors: results of a phase I study. Neurosurgery.

[138] Burgess A, Hynynen K (2016). Microbubble-assisted ultrasound for drug delivery in the brain and central nervous system. Adv Exp Med Biol.

[139] Pietrangeli D, Ricciardi G (2009). Neutral and polyanionic carboranylporphyrazines: synthesis and physico-chemical properties. Appl Radiat Isot.

[140] Ito Y, Kimura Y, Shimahara T, Ariyoshi Y, Shimahara M, Miyatake S (2009). Disposition of TF-PEG-Liposome-BSH in tumor-bearing mice. Appl Radiat Isot.

[141] Zhu Y, Koh Cheng Y, John AM, Narayan SH (2007). Recent developments in boron neutron capture therapy (BNCT) driven by nanotechnology. Curr Chem Biol.

[142] Yinghuai Z, Cheng Yan K, Maguire JA, Hosmane NS (2007). Recent developments in boron neutron capture therapy driven by nanotechnology. Boron science: new technologies and applications.

[143] Heber E, Trivillin VA, Nigg D, Kreimann EL, Itoiz ME, Rebagliati RJ (2004). Biodistribution of GB-10 (Na(2)(10)B10H10 compound for boron neutron capture therapy (BNCT) in an experimental model of oral cancer in the hamster cheek pouch. Arch Oral Biol.

[144] Bregadze VI, Sivaev IB, Lobanova IA, Titeev RA, Brittal DI, Grin MA (2009). Conjugates of boron clusters with derivatives of natural chlorin and bacteriochlorin. Appl Radiat Isot.

[145] Suzuki M, Sakurai Y, Masunaga S, Kinashi Y, Nagata K, Ono K (2004). Dosimetric study of boron neutron capture therapy with borocaptate sodium (BSH)/lipiodol emulsion (BSH/lipiodol-BNCT) for treatment of multiple liver tumors. Int J Radiat Oncol Biol Phys.

[146] Soloway AH, Zhuo JC, Rong FG, Lunato AJ, Ives DH, Barth RF (1999). Identification, development, synthesis and evaluation of boron-containing nucleosides for neutron capture therapy. J Organomet Chem.

[147] Cai J, Soloway AH, Barth RF, Adams DM, Hariharan JR, Wyzlic IM (1997). Boron-containing polyamines as DNA targeting agents for neutron capture therapy of brain tumors: synthesis and biological evaluation. J Med Chem.

[148] Menichetti L, De Marchi D, Calucci L, Ciofani G, Menciassi A, Forte C (2011). Boron nitride nanotubes for boron neutron capture therapy as contrast agents in magnetic resonance imaging at 3 T. Appl Radiat Isot.

[149] Lin WY, Chi CW, Ho YJ, Wu IC, Chung YT, Chen SD (2002). Boron-lipiodol: a potential new drug for the treatment of liver tumors. Anticancer Res.

[150] Wu G, Barth RF, Yang W, Chatterjee M, Tjarks W, Ciesielski MJ (2004). Site-specific conjugation of boron-containing dendrimers to anti-EGF receptor monoclonal antibody cetuximab (IMC-C225) and its evaluation as a potential delivery agent for neutron capture therapy. Bioconjug Chem.

[151] Zhuo JC, Cai J, Soloway AH, Barth RF, Adams DM, Ji W (1999). Synthesis and biological evaluation of boron-containing polyamines as potential agents for neutron capture therapy of brain tumors. J Med Chem.

[152] Lai CH, Lin YC, Chou FI, Liang CF, Lin EW, Chuang YJ (2012). Design of multivalent galactosyl carborane as a targeting specific agent for potential application to boron neutron capture therapy. Chem Commun.

